# Toward a functional understanding of novel fire regimes in tropical forests

**DOI:** 10.1002/ajb2.70112

**Published:** 2025-10-14

**Authors:** David Pacuk, Peter van der Sleen, Frank J. Sterck, Masha T. van der Sande

**Affiliations:** ^1^ Forest Ecology and Forest Management group Wageningen University & Research P.O. Box 342, 6700 AA Wageningen The Netherlands

**Keywords:** fire recovery, fire resilience, fire resistance, fire–vegetation feedbacks, fuel characteristics, functional traits, novel fire regimes, tree mortality, tropical forest, bosque tropical, características del combustibles, nuevos regímenes de incendios, mortalidad de árboles, rasgos funcionales, recuperación del fuego, resiliencia al fuego, resistencia al fuego, retroalimentación fuego–vegetación

## Abstract

Climate change and land‐use alterations are driving forest fires to unprecedented frequencies and intensities worldwide. Even wet tropical forests—historically rarely subjected to fire—are increasingly experiencing fire disturbances. The impact of wildfires on these forests is likely large, since many of their tree species are not adapted to fire. The extent of the consequences depends on complex feedback mechanisms between fire and vegetation, with plant functional traits playing a critical role. However, how different traits may drive fire–vegetation dynamics in such ecosystems is poorly understood due to limited consolidation of relevant data. This uncertainty leaves the future of tropical forests in question. In this review, we explore how functional traits influence the feedback between fire and vegetation. Specifically, we examine how functional traits of species shape (1) fire regimes (here considered in terms of fire frequency and intensity) through fuel characteristics, (2) their resistance to fire, and (3) their recovery after fire. Resilience is conceptualized as resistance to biomass loss and capacity for post‐disturbance biomass recovery. We provide a comprehensive overview of these traits and 12 mechanisms involved. Since the relative importance of these traits and mechanisms for fire dynamics and species’ fire resilience remains unknown, we conclude by outlining possible scenarios for how novel fire regimes might affect tropical forest resilience. We also identify knowledge gaps and avenues for future research to improve our understanding of fire–vegetation dynamics in tropical forests.

## Resumen

El cambio climático y las alteraciones del uso del suelo están provocando incendios forestales con una frecuencia e intensidad sin precedentes en todo el mundo. Incluso los bosques tropicales húmedos, históricamente poco expuestos a incendios, experimentan cada vez más perturbaciones por incendios. El impacto de los incendios forestales en estos bosques es probablemente considerable, ya que muchas de sus especies arbóreas no están adaptadas al fuego. La magnitud de las consecuencias depende de complejos mecanismos de retroalimentación fuego–vegetación, donde los rasgos funcionales de las plantas desempeñan un papel fundamental. Sin embargo, debido a la limitada consolidación de datos relevantes, se desconoce cómo las diferentes rasgos pueden influir en la dinámica entre incendios y vegetación en dichos ecosistemas. Esta incertidumbre plantea interrogantes sobre el futuro de los bosques tropicales. En esta revisión, exploramos cómo las rasgos funcionales influyen en la retroalimentación fuego–vegetación. Específicamente, examinamos cómo las rasgos funcionales de las especies configuran (1) los regímenes de incendios (considerados aquí en términos de frecuencia e intensidad de incendios) a través de las características del combustible, (2) su resistencia al fuego, Y (3) su recuperación después de un incendio. La resiliencia se conceptualiza como la resistencia a la pérdida de biomasa y la capacidad de recuperación de biomasa tras una perturbación. Ofrecemos una visión general de estas rasgos a través de 12 mecanismos. Dado que se desconoce la importancia relativa de estos rasgos y mecanismos para la dinámica del fuego y la resiliencia de las especies, concluimos describiendo posibles escenarios sobre cómo los nuevos regímenes de incendios podrían afectar la resiliencia de los bosques tropicales. También identificamos lagunas de conocimiento y vías para futuras investigaciones que mejoren nuestra comprensión de la dinámica de la relación entre incendios y vegetación en los bosques tropicales.

**Figure 1:**
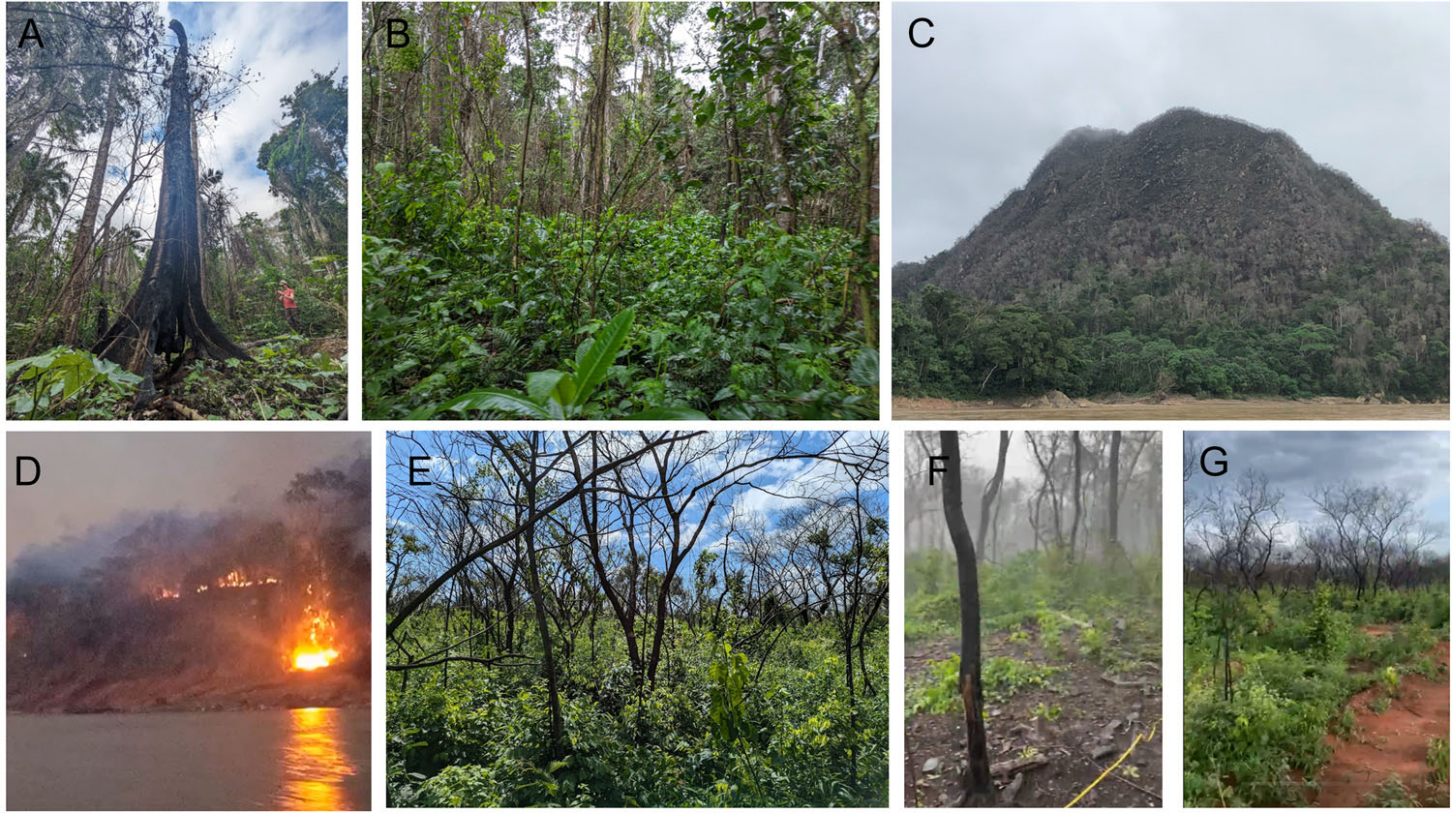
Tree mortality and resprouting after recent fire disturbances in (A, B) wet tropical forests. (C) Fully burned hilltop in Bolivian wet tropical forest and (D) the 2023 forest fires that caused it. (E–G) Mortality and short‐term resprouting in November–December 2024 after 2024 fires in Chiquitanía, Bolivia. Image credits: A–D, provided by authors; E–G, courtesy of Z. Corazza and D. Rikkers, Wageningen University & Research (used with permission).

Tropical forests are some of the most threatened ecosystems in the world, and fire is a key challenge to their conservation (Pandit et al., 2018; Ribeiro et al., 2019; Neger et al., 2024). Global fire occurrence has reached unprecedented levels, multiplying 10‐fold over the past 250 years, mainly driven by direct anthropogenic causes since the 1950s (Hantson et al., 2015; Touma et al., 2021; Sayedi et al., 2024). These unprecedented conditions are creating novel fire regimes, drastically increasing the occurrence of fire and introducing it to ecosystems that were historically nearly fire‐free (Keeley and Syphard, 2016; Rogers et al., 2020; Kelly et al., 2023; Sayedi et al., 2024). Such disturbances can push ecosystems toward alternative states at rates never previously observed (Falk et al., 2022), and tropical forests might face the same threat (Bush, 2020; Flores et al., 2024; Mata et al., 2025; see Figure 1). These forests make for some of the most important examples of threatened systems, since they contribute significantly to global biodiversity, carbon storage, and hydrological cycles—all of which are predicted to decrease or be altered unless climate change is significantly mitigated (Wright, 2010; Li et al., 2022; Sayedi et al., 2024). The loss of these forests also spells social disaster because they support the livelihoods of hundreds of millions of people, including many Indigenous communities (Wright, 2010; Lewis et al., 2015; Shuman et al., 2022). Although the exact consequences of novel fire regimes are hard to predict, even species that have evolutionarily adapted to fire can still be threatened when a regime changes (Keeley et al., 2011). Forests with some fire history are therefore vulnerable, but tropical forests, which evolved with virtually no fire, are especially at risk due to a complete lack of adaptation (Goldammer, 1990; Kauffman and Uhl, 1990; Slik et al., 2010; Neger et al., 2024). As wildfires become a structural issue in these systems, the impact of these novel conditions ultimately depends on the extent to which these ecosystems can resist fire damage and can recover after fire. This resistance and recovery, in turn, has been shown to greatly depend on a suite of traits of the species involved in many different ecosystems (Enright et al., 2014; Clarke et al., 2015; Foster et al., 2018; Tangney et al., 2020; Harrison et al., 2021; Rodman et al., 2021; Falk et al., 2022; Lamont, 2022).

**Figure 2:**
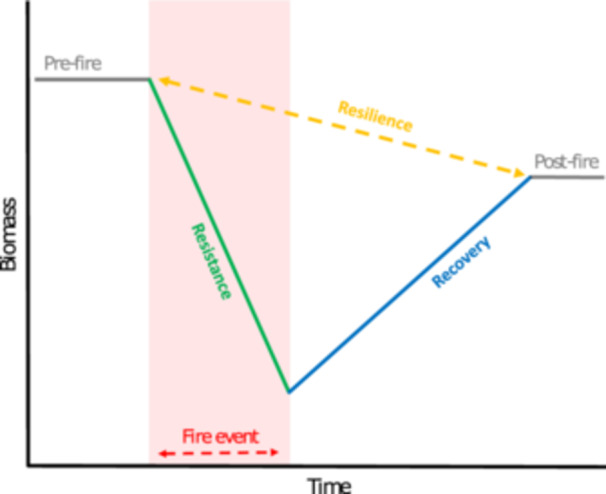
The resistance–recovery–resilience (RRR) framework (Lloret et al., 2011), adapted to fire‐based disturbances and applied at the level of individual trees. Each phase is defined as loss or gain of (living) biomass over time (e.g., changes in growth, mortality, and recruitment). Resistance is the inverse of biomass loss during a fire event, and recovery is the post‐disturbance increase in biomass relative to the minimum after the fire event (i.e., return to pre‐fire baseline). Although these processes can result in complications like arrested or deflected succession at the ecosystem level, our focus on individual organisms allowed us to simply express resilience in terms of biomass. Resilience is thus defined by the difference between pre‐ and post‐fire biomass over time. The duration of a fire event can be equated to the residence time of any single fire event.

Understanding the impacts of novel fire regimes on forest vegetation requires unravelling the effects of their intricate feedbacks (Bond et al., 2005; Archibald et al., 2013). Central to these feedbacks are the relevant species’ functional traits: morphological, physiological, phenological, or behavioral features measured on organisms that can be linked to their performance (Violle et al., 2007). These traits determine the dynamics on both ends of the fire–vegetation feedback: how vegetation shapes fire regimes by determining fuel characteristics and how fire impacts vegetation based on their fire resilience. Here, we define fire resilience based on two components (Figure 2): resistance (i.e., the direct impact of the disturbance on living biomass) and recovery (the post‐disturbance return of living biomass from the minimum to a reference baseline determined from undisturbed controls) (Lloret et al., 2011). The impact of fire is thus measured at an individual level through changes in (living) biomass due to fire, as mediated by species resistance and recovery. The use of functional traits to describe plant strategic axes has proven fruitful for understanding global vegetation dynamics and ecosystem processes (Gibert et al., 2016). However, numerous groups have noted the lack of empirical work on the larger suite of trait‐based mechanisms that drive fire–vegetation feedback dynamics, especially beyond well‐known fire‐adaptation traits (Brando et al., 2012; Archibald et al., 2018; Hood et al., 2018; Fernández‐García et al., 2020; McLauchlan et al., 2020; Staver et al., 2020; Popović et al., 2021; Shuman et al., 2022; Maillard, 2023). This lack of empirical work leads to a poor understanding of which traits shape fire regimes and fire resilience (i.e., resistance and recovery) and is the limiting factor to reliably model and predict future fire occurrences and impacts (Archibald et al., 2013; McLauchlan et al., 2020; Stevens et al., 2020; Sayedi et al., 2024; Schwilk et al., 2025 [in this issue]).

## Scope of the review

Many functional traits relevant to fire resilience have been studied primarily in fire‐adapted species outside the tropics, with limited synthesis across traits. While previous studies have synthesized these traits into frameworks of flammability (Popović et al., 2021), community fire resilience (Falk et al., 2022), and species‐level climate change resilience (Kühn et al., 2021), the mechanisms underlying tree fire resilience remain elusive. Our review is thus aimed to build a unified trait‐based framework linking functional traits to species‐level fire resilience at the individual level of organization. With this aim in mind, we only considered individual and species functional traits. We excluded related response and effect traits (e.g., flammability traits; see Lavorel and Garnier, 2002; Schwilk et al., 2025 [in this issue]), which describe species contributions to fire regimes or their ecological responses to them, rather than the physiological and morphological mechanisms that underpin fire resilience (see also Violle et al., 2007). We also did not consider higher levels of organization (e.g., communities, ecosystems). The role of environmental variables in shaping fire regimes is also beyond the scope of this review and has been discussed and summarized elsewhere (e.g., Fernández‐García et al., 2020; Rogers et al., 2020; Wasserman and Mueller, 2023; Scholten et al., 2024). While we focused on tropical forests, our sources come from a wide variety of ecological settings and may also prove relevant outside of our scope. With that in mind, our theoretical and mechanistic approach establishes a crucial framework for advancing future analyses of forest fire resilience, particularly by addressing the often‐overlooked role of vegetation ecology, with a specific focus on tropical ecosystems. We aimed to determine how individual‐ and species‐level functional traits shape (1) fire regimes, and individual‐ and species‐level (2) fire resistance and (3) fire recovery. We do so by providing a synthesis based on a qualitative literature review of 164 peer‐reviewed scientific publications (see Appendices S1, S2). We then identify and discuss implications for further research on community‐level fire resilience.

## A FUNCTIONAL TRAIT‐BASED FRAMEWORK

**Figure 3:**
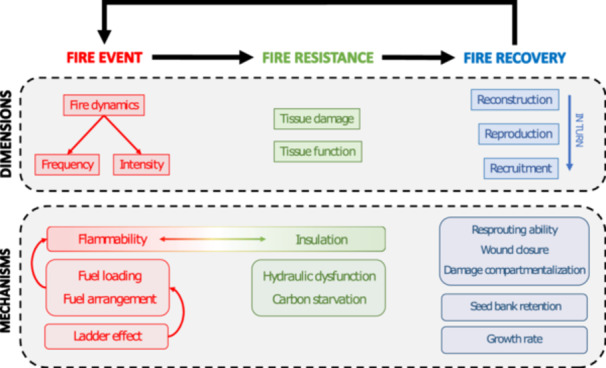
Conceptual model for the mechanistic workings of the three components of fire resilience. Each component of the fire resilience process (fire event, resistance, and recovery) is further split into the different dimensions that define them, with the most important mechanisms listed below. The workings of these mechanisms are based on functional traits (see overview in Figure 4).

**Figure 4:**
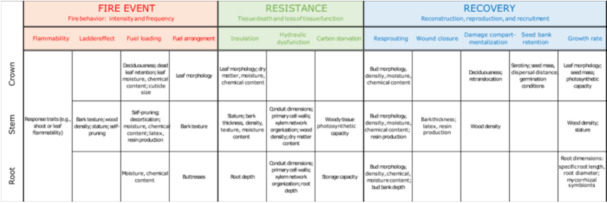
Summary of relevant traits for each of the mechanisms as part of the three components of fire resilience (fire event, resistance, and recovery: see Figure 3; summary of each trait and literature sources: Appendix S1).

Due to the feedback between fire and vegetation, species traits impact both the fire itself and tree fire resilience. We therefore split the framework into three components: the fire event, fire resistance, and fire recovery (Figures 3, 4). A fire event is described by the fire behavior, that is, how it reacts to the influences of fuel, weather, and topography (McLauchlan et al., 2020). A probabilistic estimate of such fire behavior compared to relevant conditions (e.g., topography, edaphically mediated landscape wetness gradients, climate and weather conditions, related weather events) defines a fire regime (McLauchlan et al., 2020), making it categorizable based on syndromes (or “pyromes”) of fire frequency and intensity (Archibald et al., 2013). Here, we specifically focus on the impact of fuel type, which depends on tree functional traits. While the concept of a fire regime and its defining dimensions is theoretically complex and subject to debate (Keeley, 2009; Krebs et al., 2010), we define it here in terms of fire intensity and frequency because these dimensions are most directly influenced by vegetation traits and trait–fire feedbacks (Archibald et al., 2013). We use “fire dynamics” to refer broadly to these two dimensions of fire regimes. Fire intensity is the rate of energy released (Archibald et al., 2013) or the amount of energy released per unit time per unit area (McLauchlan et al., 2020). A fire's intensity is thus measurable through a combination of temperature, residence time, and radiant energy (Keeley, 2009). Fire frequency constrains fire intensity by creating a fuel‐limited system (McLauchlan et al., 2020), whereas low frequency would allow for more variability in intensity (Archibald et al., 2013). Traits impact the intensity and frequency of a fire by modifying its fuel (i.e., living and dead plant material), thereby shaping both the fire's initial conditions extrinsic to the tree (e.g., litter availability) and the impact of fire on the tree itself. As such, functional traits are mechanistically central to the feedback dynamic between fire and vegetation.

Fire resistance and recovery determine how an individual or species fares during and after the fire event. Resistance to a fire is quantifiable in two main ways. First, tissue death leads to the direct loss of living biomass. It is thus primarily a matter of heat insulation to prevent damage to the phloem, cambium, and xylem. After all, temperatures exceeding 60°C can lead to tissue mortality (Michaletz and Johnson, 2007), and even lower temperatures can suffice if applied for longer periods of time (Bär et al., 2019). Second, extreme temperatures can also lead to the loss of tissue function, and thereby of key physiological functions. For example, an often‐overlooked aspect of tree mortality to fire is hydraulic dysfunction due to abrupt local drought conditions (Hood et al., 2018). Species recovery refers to processes of recuperation after a fire and can be defined in three ways: reconstruction (to reverse damage done), reproduction (to produce new offspring), and recruitment (growth of new seedlings to adulthood). The last two are relevant for species‐level recovery but not for individual‐level recovery.

### The fire event

#### Flammability

Traits shape fire behavior through flammability, which is in turn determined by three main mechanisms: ladder effects, fuel loading, and fuel arrangement (see Figure 3). While we consider flammability not as a functional trait but as a mechanism characterized by certain response or effect traits, functional traits do influence flammability by determining the ability of a fuel to burn (Varner et al., 2015; Archibald et al., 2018; Schwilk et al., 2025 [in this issue]). Flammability is commonly described through features such as ignitability (the delay on ignition), sustainability (the duration of combustion), and combustibility (the mass loss rate) (Blauw et al., 2015; Varner et al., 2015; Popović et al., 2021). These properties are frequently analyzed and applied in experimental settings (e.g., Kane et al., 2008; Bauer et al., 2010; Blauw et al., 2015; De Magelhães and Schwilk, 2021), but are rarely quantified for natural fires. Higher ignitability would lead to higher fire frequencies, while high combustibility would increase energy release and consequent fire intensity. Recent assessments show that flammability can reliably be explained by the functional traits that comprise a fuel load (Varner et al., 2015; Pausas et al., 2017; Santacruz‐García et al., 2019; Popović et al., 2021). However, flammability always depends on fuel characteristics. Although many studies have focused on quantifying flammability through response traits (e.g., Wyse et al., 2016, 2018; Santacruz‐García et al., 2019; Alam et al., 2020; Ortiz et al., 2023), here we try to identify the functional traits that mechanistically explain flammability across the entire tree.

#### Ladder effects

“Ladder effect” refers to the vertical spread of a fire, from the surface (i.e., a litter layer or understory) to the canopy. At the extremes of this verticality spectrum, a litter fire represents low verticality, whereas a canopy fire exemplifies high verticality. This vertical spread of fire into a canopy often depends on the availability of so‐called ladder fuels, most commonly either vines (Leicht‐Young and Pavlovic, 2015) or lower non‐canopy branches of a tree (Bowman et al., 2014; Archibald et al., 2018), which are used as a means to ascend trees. The likelihood of vine infestation is most importantly determined by bark texture, since rougher barks allow for easier ascent (van der Heijden et al., 2008), but may also depend on tree wood density and stature because the crowns of taller and fast‐growing trees are more likely to have a vine infestation due to the greater light availability (van der Heijden et al., 2008; Sfair et al., 2016; Reis et al., 2020) (see Figure 4). However, infestation of a crown does not always imply a ladder effect because vines can also grow from one crown to another. The presence and height of lower subcanopy branches are largely determined by a tree's size and the ability of a species to self‐prune (Bowman et al., 2014; Archibald et al., 2018), which is often seen as a strategic adaptation in fire‐prone environments (Pausas et al., 2017). Hence, the vertical spread of a fire can be influenced by architectural traits and their contributions to vine infestation. The levels of verticality of a fire event that arise from ladder effects can in turn affect how other traits contribute to fire dynamics. For example, deciduousness may enhance survival during crown fires by reducing canopy fuel loads (i.e., flammability at higher strata), but it can simultaneously increase fire frequency or intensity by contributing to the fuel load at the surface, potentially reinforcing surface fire spread and vertical fuel connectivity. Recognizing fire verticality as a structural dimension is thus a prerequisite for assessing an ecosystem's fuel dynamics because it is defined by the fuel load and arrangement.

#### Fuel loading

Fuel loading refers to the quantity and quality of standing and lying organic material, which can potentially increase fire intensity and frequency (see Figure 3). It is positively related with deciduousness (de Magalhães and Schwilk, 2021), dead leaf retention (Bowman et al., 2014), self‐pruning (de Magalhães and Schwilk, 2021), and decortication (Gagnon et al., 2010; Bowman et al., 2014) (see Figure 4). All these traits represent higher leaf, branch, and bark turnover rates, which lead to higher fuel loading. The occurrence of these traits differs across fire regimes. For example, self‐pruning is expected to occur only in fire regimes where canopy fires are common (Falk et al., 2022) and might not occur for pyrogenic (or self‐immolating) species (Gagnon et al., 2010). Similarly, deciduousness and dead leaf retention have very different strategic values depending on whether fire typically reaches the canopy. On one hand, the retention of (dead) leaves can increase crown flammability (Bowman et al., 2014) and can leave a tree vulnerable to heat plumes (Midgley et al., 2011; Meza et al., 2023). On the other hand, retention reduces the size of a litter layer, reducing the frequency and intensity of understory fires. Additionally, desiccated leaves serve as rapidly burning fuels, promoting fast‐burning fires that are deemed beneficial in protecting belowground organs (Gagnon et al., 2010).

Fuel loading is further affected by moisture and chemical contents of all litter types (e.g., leaf, bark, branch), which can change the litter decomposition rates (Varner et al., 2015; de Magalhães and Schwilk, 2021) and lead to complex interactive effects on litter flammability (“non‐additive effects”, as reviewed by Blauw et al., 2015). Diverse plant chemicals can increase flammability (Schwilk et al., 2025 [in this issue]): Calcium and magnesium can increase flaming durations; nitrogen, phosphorous, and tannin concentrations increase flame and smoulder durations; terpenoids can have a variety of contradictory effects on flammability (Popović et al., 2021); volatile organic compounds (VOC) are known to reduce ignition temperatures (Bowman et al., 2014); isopropenoids and essential oils can be highly flammable (Parsons et al., 2015). Additionally, latex and resins can have positive effects on flammability because they are likely potent combustibles (Barlow et al., 2003a). Furthermore, the cuticle of leaves can help retain moisture, potentially decreasing flammability (de Magalhães and Schwilk, 2021).

#### Fuel arrangement

Fuel arrangement refers to the composition and distribution of fuel, that is, differences in types of fuel and their morphology: shape (e.g., dimensions, structure, particle size), physical composition (e.g., particle density, chemical composition), and spatial arrangement (e.g., bulk density) (Bowman et al., 2014; Blauw et al., 2015; Schwilk, 2015, 2025 [in this issue]; Varner et al., 2015; de Magalhães and Schwilk, 2021) (see Figure 4). These can all further affect the flammability of a canopy or litter layer by defining its levels of aeration and moisture retention (Blauw et al., 2015). Three main groups of traits influence fuel arrangement. First, leaf morphology, where long, curly leaves will be better aerated and retain less moisture, increasing both ignitability and combustibility (Bowman et al., 2014; Parsons et al., 2015; Varner et al., 2015). Similarly, bark texture can be logically assumed to affect aeration and moisture retention in a litter layer because rougher barks have more fissures and can be flakier (Barlow et al., 2003a). Additionally, the presence of buttresses can increase the accumulation of litter underneath a tree, thereby facilitating much higher intensities when fire strikes (Barlow et al., 2003a).

### Fire resistance

Fire resistance can be quantified by tissue death and loss of tissue function (see Figure 3). Tissue death refers to the decline of living biomass during a fire event, meaning direct mechanical damage suffered from fire. It is often quantified as tree mortality or species population decline, but can also refer to burned parts of individual trees. Loss of tissue function instead is the indirect loss of living biomass due to the failure to maintain basic physiological processes. These two dimensions have also been respectively named first‐ and second‐order effects (Bär et al., 2019). For the first dimension, the degree of tissue death depends on how well a tree is insulated from heat and fire. “Insulation” is the conceptual opposite of flammability: a tree's ability to *not* burn and to avoid heat stress from damaging the cambium, phloem, and xylem. A low insulation capacity is thus in most cases associated with a high capacity to burn, each having their own strategic value (Pausas et al., 2017).

#### Insulation

Bark thickness and tree stature (size) is the classic combination of traits that consistently have been found to be central to fire resistance through insulation (Bauer et al., 2010; Brando et al., 2012; Clarke et al., 2013; Oliveras et al., 2014; Rosell, 2016; Pausas, 2017; Kühn et al., 2021; Falk et al., 2022; Mostacedo et al., 2022; Meza et al., 2023) (see Figure 4). Bark thickness is the strongest indicator of insulative capacity because bark is the main barrier against heat transfer toward the cambium, and it strongly increases with tree size (Rosell, 2016; Pausas, 2017; Mostacedo et al., 2022). Because of this association with tree size, relative bark thickness (i.e., bark thickness relative to bole diameter; Midgley and Lawes, 2016) provides a fitting mechanical basis for quantifying and comparing fire resistance between life stages, species, and populations (e.g., Lawes et al., 2013; Pausas, 2015; Lawes et al., 2021). Additionally, since insulation is primarily a function of outer bark rather than inner bark, interpretations of bark thickness as a fire resistance trait would ideally account for this distinction (Rosell, 2019).

Aside from affecting bark thickness, stature may shape fire resistance in a non‐insulative way. According to the escape height hypothesis, a taller tree will have a safer crown, preventing tissue death in the canopy (Midgley et al., 2011; Michaletz et al., 2012; van der Sande et al., 2023). In contrast, for a fire‐resister tree species, larger trunk diameter, not height, is correlated with less mortality (Midgley et al., 2011). This effect of tree diameter might be due to fires not being able to span the entire circumference of a tree, and the question remains in which specific contexts this outweighs the effect of tree height. Similarly, an increase in stature also increases root depth, which in turn increases the depth of the soil layer protecting and insulating roots from fire (Michaletz and Johnson, 2007).

Bark density, structure, and moisture content are also considered important for tree insulation (Bauer et al., 2010; Brando et al., 2012; Poorter et al., 2014; Rosell et al., 2014; Falk et al., 2022) (see Figure 4). However, their interrelatedness complicates interpretation of their effects, and experimental results are contradictive. On one hand, bark density relates negatively to heat transfer rates (Brando et al., 2012); on the other, species with higher bark densities reach lethal temperatures (≥60°C) faster (Bauer et al., 2010). Bauer et al. (2010) argued that the greater air spaces at lower densities provides additional insulation, which is also why coarser (i.e., more fissured) bark structures increase insulation. This kind of porosity also enables higher moisture contents, which are shown to be an important heat sink due to the moisture that caps heat transfer at 100°C (the point at which water changes from liquid to gas). Because this moisture also absorbs and diffuses a lot of energy during evaporation, more time is needed to reach lethal temperatures (Bauer et al., 2010; Brando et al., 2012). Still, water is also highly conductive of heat, which is why higher bark densities (and lower moisture content) might also lead to positive effects (Brando et al., 2012). While bark coarseness and moisture content are expected to increase with bark thickness, these traits come at a trade‐off with bark density (Barlow et al., 2003a; Poorter et al., 2014; Rosell et al., 2014). In all, these complex interactions can lead to mixed results, so that bark density is often considered less important for fire resistance than bark thickness (Michaletz and Johnson, 2007; Bauer et al., 2010; Brando et al., 2012). However, there may be an optimum for fire resistance in this thickness–density trade‐off (Poorter et al., 2014).

In the crown, the amount of insulation is determined by leaf morphology and leaf, branch, and twig dry matter and their moisture level, and chemical content (see Figure 4). Morphology works similarly here as it does in a litter layer, with longer, curling and lobed shapes allowing for better aeration and retaining less moisture, and thus allowing for more local heat transfer, igniting more easily, and burning more intensely and rapidly (Bowman et al., 2014; Parsons et al., 2015; Varner et al., 2015). Generally, a higher surface‐to‐volume ratio and specific leaf area (SLA) increases ignitability (Bowman et al., 2014; Popović et al., 2021), especially when moisture contents are low (de Magalhães and Schwilk, 2021). It is generally accepted that finer (i.e., lower diameter) leaves are more flammable in open crowns, while thicker and coarser leaves will be better insulated (Bowman et al., 2014). For branches and twigs, the basic principle revolves around leaf dry matter content and spatial dimensions, since more dry matter allows faster fire propagation in the right conditions (Santacruz‐García et al., 2019; Potts et al., 2022; Ortiz et al., 2023). Moisture content is an important constraint on flammability (or, insulator) because more energy is required for ignition and sustained combustion (Bowman et al., 2014). Additionally, while chemical contents usually increase flammability, many types can reduce it at times (as reviewed by Popović et al., 2021; Schwilk et al., 2025 [in this issue]). Lastly, across the whole tree, latex and resin production can have similarly contradictive effects; they can be a potent combustible and act as insulators (Barlow et al., 2003a).

#### Hydraulic dysfunction

Loss of tissue function is the second dimension of fire resistance (see Figure 3). It impacts individual and species resistance through two indirect consequences of fire: hydraulic dysfunction and carbon starvation, which are both often‐overlooked causes of tree mortality from fire. First, the vascular system can become dysfunctional when fire causes spontaneous local atmospheric droughts (Hood et al., 2018). Fires lead to higher temperatures, that cause higher vapor pressure deficits (VPD), and thus higher transpiration demands on trees (Choat et al., 2018). Excessive water loss in response to high VPD brings the risk of xylem cavitation (formation of gas emboli; Choat et al., 2018; McDowell et al., 2022). Cavitation resistance generally depends on conduit dimensions (i.e., length and diameter), porosity of the primary cell walls (i.e., pit membrane thickness and pore size), and xylem network organization (i.e., types of conduits; see Figure 4) (Markesteijn et al., 2011a, 2011b; Choat et al., 2018). Based on these traits, each species is expected to develop different sensitivities to xylem pressure (Choat et al., 2018), with especially low sensitivities in wet tropical forest species (Maherali et al., 2004).

Traits that enhance cavitation resistance by being correlated with the above cell‐level traits include wood density (Markesteijn et al., 2011b) and stem dry‐matter content (Poorter and Markesteijn, 2008). Additionally, deciduousness increases cavitation resistance because a tree without leaves will experience less evapotranspiration in a heat plume (Midgley et al., 2011). Furthermore, root depth is essential to maintain a desirable water supply and prevent cavitation (Lopez‐Iglesias et al., 2014; Freschet et al., 2021). In addition to cavitation, xylem deformation can also cause hydraulic dysfunction (Hood et al., 2018; Bär et al., 2019). However, the cellular mechanisms underlying the deformation have not been elucidated, and empirical evidence is ambiguous (Michaletz et al., 2012; West et al., 2016; Bär et al., 2018; Partelli‐Feltrin et al., 2023). As such, xylem deformation has not yet been clearly linked to tree functional traits (reviewed by Bär et al., 2019).

#### Carbon starvation

A plant can become starved of carbon when phloem and cambium necrosis leads to blockage of carbon translocation or when resource acquisition tissues (typically foliage) are killed or otherwise critically impaired. Each of these occurrences affect tree‐wide functionality (e.g., stunted growth) and potentially cause mortality (reviewed by Hood et al., 2018; Bär et al., 2019). These impairments are especially relevant when the circumference of a tree is heated (almost) entirely, with effects equivalent to those of girdling (Noel, 1970; Ryan, 2000). Regaining functionality of the phloem can take years or even decades (Smith et al., 2016; Stambaugh et al., 2017), unless the loss of the cambium was too severe for recovery (Bär et al., 2019). Carbon starvation can even feed back into drought‐related mortality because a lack of carbon availability can stop fine root production (Marshall and Waring, 1985), seriously reducing water uptake and raising the risk of premature stomatal closure and/or cavitation (Tyree and Zimmermann, 2013). Few functional traits can directly prevent carbon starvation beyond the previously discussed insulation traits. However, higher storage capacity in relevant tree components (see Furze et al., 2019) may help avoid or delay total starvation. Additionally, photosynthesis in woody tissue can provide photosynthates to the roots by recycling respired CO_2_ concentrated in the woody tissues (Pfanz, 2008; Ávila et al., 2014; Vandegehuchte et al., 2015). This capacity for photosynthesis in turn depends on stem and bark chlorophyll content, C/N concentrations, densities, and specific areas of the stem and bark (Ávila et al., 2014, 2020; Rosell, 2019). Research shows that woody stem photosynthesis can be proportional to leaf photosynthesis for some scrub species (Ávila et al., 2020), but whether woody tissue photosynthesis is common and comparable to that in tropical forest trees remain unknown.

### Fire recovery

#### Resprouting

Fire recovery captures processes that facilitate the recovery of an individual or species after fire. Post‐fire recovery consists of three common phases: reconstruction, reproduction, and recruitment. First, reconstruction concerns the individual‐level recovery from any damage suffered during fire. One of the most prominent reconstruction mechanisms in this setting resprouting, which relies on the ability to develop, protect, and source a viable bud bank (Clarke et al., 2013). Any part of a tree—its crown, stem, and roots—can resprout (see Figure 4). Investment in the ability to resprout often comes at the cost of resistance traits (Vesk, 2006) and implies a trade‐off with beneficial reproductive traits such as serotiny and seed output because investments go to a tree's bud bank instead (Clarke et al., 2013; Kühn et al., 2021). Successful post‐fire resprouting relies on the protection of the buds during fire and hence is strongly associated with fire resistance traits. Bud protection can generally be achieved in two ways: through a non‐flammable strategy, where protective traits (e.g., bark‐ and leaf‐based resistance traits) help guard the buds (Clarke et al., 2013), or a fast‐flammable strategy, where fires are kept fast and at low intensities (Pausas et al., 2017). The last is considered especially advantageous for smaller trees, since they have a higher probability to resprout from their base and a faster fire would transfer less heat through the soil (Mostacedo et al., 2022). Because smaller trees can also be assumed to have a thinner bark, a non‐flammable strategy is viable. Both strategies give resprouters a higher probability to survive and recover after frequent fires (Clarke et al., 2009), forming a “persistence niche” of species that increase in abundance after repeated fires (Clarke et al., 2013).

Additionally, since bud survival is essential, resprouting success is known to depend on bud heat tolerance, as determined by bud morphology, density, and moisture content (Bär et al., 2021; McClure et al., 2022) (see Figure 4). As with bark properties, buds that are larger in volume and diameter, less dense, and contain more moisture will transfer less heat to their cores due to the greater internal accumulation of air, leading to higher survival probability (Bär et al., 2021). Resin production and chemical content (e.g., secondary metabolites) in the buds and surrounding tissues may also increase bud heat tolerance by increasing cellular thermal stability, but empirical evidence remains equivocal (Bär et al., 2021). For root resprouters specifically, bud bank depth is another important positive insulation factor due to the insulative quality of soil (Freschet et al., 2021).

#### Wound closure and damage compartmentalization

Two other reconstruction mechanisms are wound closure and damage compartmentalization (see Figure 3). Wound closure rate refers to the area of wound surface closed over time. The efficiency of wound closure increases with bark thickness because widely dilated parenchyma rays support the closure process (Romero and Bolker, 2008). Species with higher wood density are also more likely to produce latex and resin, which can seal wounds and deter insects (Poorter et al., 2014). Damage compartmentalization avoids the spread of damage to nearby tissue and increases with wood density because the thick‐walled cells of denser woods can limit the spread of decaying tissue (Romero and Bolker, 2008). Additionally, when a crown needs to recover, favorable traits include deciduousness, because crown restoration is a regular occurrence, and retranslocation, that is, the resorption and remobilization of constituents from scorched foliage to living branches (Varner et al., 2021).

#### Seed bank retention

Reproduction, an important form of fire recovery at the species level (see Figure 3), depends on the retention of a seed bank, the production of new seeds, and the dispersal syndromes of the species. The success of seed bank retention depends on several factors. The typical fire‐related dispersal syndrome is serotiny, where seeds are released in response to fire. Serotiny is considered part of a hot‐flammable strategy, in which a high‐severity fire is facilitated and a niche is constructed for the serotinous species to reproduce as successfully as possible (Pausas et al., 2017; Falk et al., 2022; Keeley and Pausas, 2022). However, since serotiny is an evolutionary adaptation to fire, it is only rarely encountered in the tropics, specifically in dry (sub)tropical ecosystems (Skoglund, 1992; Williams et al., 1999; Lamont et al., 2020). Successful dispersal, regardless of syndrome, is typically correlated with traits such as seed mass, dispersal distance, and germination conditions (see Figure 4). Seed mass is typically lower for serotinous species and is considered beneficial when fire is more common and quick colonization is desirable (Fernández‐García et al., 2020; Van der Sande et al., 2023). In comparison, seed mass is higher for species that resprout or have seedlings that grow rapidly because more resources are required (Green and Juniper, 2004). Dispersal distances are typically higher where fire is less frequent (Safford and Stevens, 2017) and vice versa (Falk et al., 2019). Germination in fire‐adapted species is typically either heat‐ or smoke‐driven (Fernández‐García et al., 2020; Falk et al., 2022). Aside from these fire‐specific traits, relevant reproductive traits can be understood through general theories on post‐disturbance colonization (e.g., Cadotte, 2007; Brown and Boutain, 2009). For example, the disperser community in a post‐fire ecosystem may be less abundant and diverse, which may favor the recovery of wind‐dispersed species (Hammill et al., 1998).

#### Growth rates

Certain species traits increase the percentage of seedlings that survive into adulthood after a fire, that is, “recruitment” (see Figure 3). Recruitment relies primarily on high growth rates. A higher potential growth rate is more beneficial for two reasons. For one, post‐fire understory light levels are generally high, favoring fast‐growing, light‐demanding species. For another, higher frequencies of fire shorten the effective growth period (Archibald et al., 2013), thus decreasing survival probability and plant species diversity (Swanson et al., 2011). Key traits for fast growth include leaf morphology, wood density, stature, seed mass and photosynthetic capacity (Poorter et al., 2008; Paine et al., 2015; Gibert et al., 2016) (see Figure 4). These traits vary along a fast–slow plant economics spectrum, defined by the trade‐off between tissue construction costs and growth rates (Díaz et al., 2016; Gibert et al., 2016). Leaf dimensions, seed mass, and photosynthetic capacity all dictate resource availability and acquisition, while wood density affects mechanical robustness. With one coming at the cost of the other, a faster growth strategy can thus be considered high risk, high reward, resulting in higher likelihood of mortality (Poorter et al., 2008). As ecosystems become more fire‐prone, how will this trade‐off hold? While slow‐growing species will likely attain higher maximum fire resistance in their lifetime, fast‐growing species may develop relatively thick bark at an earlier stage in life thanks to their size, potentially guaranteeing survival into adulthood.

This logic is generally considered to apply in parallel to the roots, where root dimensions would change accordingly; a fast‐growing tree generally has a high specific root length (total length/dry biomass) with small root diameter, and vice versa (Bergmann et al., 2020). This investment into root length over diameter similarly allows for faster resource acquisition with minimal biomass investment. However, the ability to form mycorrhizal symbioses—particularly with arbuscular fungi—can significantly alter nutrient uptake in ways that break with the fast–slow continuum. Evidence indicates that thick, unbranched roots (i.e., slow strategies) benefit most from such collaboration (de Vries et al., 2021; Weemstra et al., 2023). Thus, this ability to establish mycorrhizal symbioses is another key trait to consider when evaluating acquisition strategies for belowground resources and their contributions to fire resilience (Hartnett et al., 2004; Hart et al., 2005; Atala et al., 2023).

## DISCUSSION

Functional traits provide a mechanistic link to understand how vegetation shapes fire regimes and to understand species resistance to and recovery from fire. In our review of 164 peer‐reviewed publications, we detailed traits that were found or expected to be important for fire dynamics and species resilience (i.e., resistance and recovery) in tropical forests and identified 12 beneficial mechanisms. Considering these traits and mechanisms, we discuss the impact that future fires may have on tropical forests. Since few data are available to quantify the relative importance of these traits and mechanisms, we conclude by outlining future research avenues.

### The future of tropical forests

In a future where fires are generally expected to become more frequent and intense, we expect both dry and wet tropical forests to drastically change. Increases in fire frequency and intensity can lead to filtering for pyrophytic species and traits during regeneration (Araújo et al., 2017; Nolan et al., 2021). While increased fire intensity may lead to an increased abundance of species with fire resilience traits (Figure 4), it could also simply increase the abundance of fast‐growing species that favor disturbed conditions. However, as fire frequency increases and fire return intervals shorten, these fast‐growing pioneers may instead prove too vulnerable, and conditions may again favor species with fire resilience traits instead. Hence, the impact of fires on the future of tropical forests remains highly uncertain and will depend strongly on the interaction between fire frequency and intensity. In extreme cases, novel conditions may push ecosystems past tipping points, leading to critical transitions of forest ecosystems (Fletcher et al., 2020; Flores et al., 2024). For example, the loss of tropical forest due to fire may lead to reductions in evapotranspiration and precipitation, creating droughts and further exacerbating the already extreme conditions of novel fire regimes (Brando et al., 2014). Such feedback loops between changes in trait compositions and environmental conditions may thus turn wet tropical forests into dry ones or even lead to collapse of tropical forest ecosystems into persistently degraded systems or savanna‐like landscapes (Warman and Moles, 2009; Brando et al., 2014; Flores et al., 2024; Williamson et al., 2024). As such, the potential for alternative stable states must be a central consideration in future resilience frameworks, particularly when linked to species‐level traits.

### Future research

We identified four main avenues for further research to determine the relevance of these traits in the context of novel fire regimes of tropical forests.

#### Experimental studies

Although many traits have been associated with species‐specific differences in fire resistance, the physiological and morphological changes in a tree during fire and the ultimate causes of fire‐induced tree mortality often remain unknown (Hood et al., 2018). Experimental studies to quantify of tree responses to fire may help link functional traits to relevant response traits and address a variety of important knowledge gaps. Recent research on leaf and shoot flammability have already established critical links between functional traits such as leaf and shoot architecture, and flammability traits (e.g., Potts et al., 2022; Alam et al., 2020, 2024). Further progress in linking functional traits to flammability from a whole‐tree perspective could prove highly valuable in predicting fire resilience at higher levels of organization.

We see several avenues for such experimental fire research. First, the roles of different plant chemicals (including latex and resin) in flammability are often mentioned (e.g., Barlow et al., 2003a; Bowman et al., 2014; Parsons et al., 2015; Popović et al., 2021), but their high diversity and interrelatedness make their effects on fire resilience challenging to untangle. More specialized chemical analyses combined with flammability experiments could help to move past this categorical simplification. Second, the insulation effect of bark density is still highly entangled with that of many other bark traits (e.g., moisture content, dry‐matter content, thickness, texture), and how bark density improves insulation is still topic of debate (Bauer et al., 2010; Brando et al., 2012; Poorter et al., 2014; Rosell et al., 2014). Third, xylem deformation has gained increasing attention over the past decade, but empirical evidence of the process and its significance to a tree's fire resilience remain elusive (Michaletz et al., 2012; West et al., 2016; Bär et al., 2018; Partelli‐Feltrin et al., 2023).

#### Long‐term monitoring

Second, long‐term monitoring studies are needed to understand not only species resistance, but also traits that determine their longer‐term recovery after fire. This information is important for determining delayed mortality (Barlow et al., 2003b; Berenguer et al., 2021) and to assess how species and ecosystems can recover toward pre‐disturbance states. Although the long‐term recovery of tropical forests after major disturbances has been studied (e.g., Poorter et al., 2021; Van der Sande et al., 2024), how traits steer long‐term recovery after fire remains unknown.

#### Climate and environmental gradients

Third, trait‐based studies of fire resilience in the context of climate and environmental gradients are needed because climate and fire regime are tightly connected and both can filter the traits that are present in a community (e.g., Fernández‐García et al., 2020; Rogers et al., 2020; Wasserman and Mueller, 2023; Scholten et al., 2024). While some traits may be important for fire resilience in drier and more fire‐prone ecosystems, other traits may drive fire resilience in wetter ecosystems. We hypothesize that adaptation to fire in dry tropical forests mainly happens by resprouting, whereas species in wet tropical forests will most likely show fire resilience mainly through higher growth rates and stature‐based fire resistance.

#### Larger organizational, spatial, and temporal scales

Better understanding of the role of traits for tree‐level and species‐level fire resilience will allow us to better understand fire resilience across phylogenetic groups and across tree communities. Furthermore, it will allow a spatial and temporal scale up using remote sensing and mechanistic modelling to predict future fire resilience across the tropics. We currently lack models that include vegetation traits in their projections of fuel and fire characteristics (Archibald et al., 2018). Additionally, earth system models are still unable to simulate dynamic vegetation cover, which is key to assess tipping points (Flores et al., 2024). Working toward such levels of modelling would require realistically parameterized and well‐validated estimates of the involved physiological processes and traits (Zuidema et al., 2013). The development of these foundations would enable great leaps in the prediction of fires and their impacts in a fire‐prone future, thus aiding global conservation of the tropical forests on which we rely.

## AUTHOR CONTRIBUTIONS

The idea for the paper was conceived collectively in response to a call for papers by the *AJB*. D.P. reviewed literature, designed the manuscript, and wrote drafts. All authors helped interpret results, reviewed design and contents, and approved the final version of the manuscript.

## Supporting information


**Appendix S1.** Summary of data on fire resilience traits and literature sources.

**Appendix S2.** Summary of data on fire variables and literature sources.

## Data Availability

No new data were used for this review.
